# Bound Ca^2+^ moves faster and farther from single open channels than free Ca^2+^

**DOI:** 10.3389/fphys.2023.1266120

**Published:** 2023-12-20

**Authors:** S. L. Mironov

**Affiliations:** Institute of Neuro- and Sensory Physiology, Georg-August-University, Göttingen, Germany

**Keywords:** calcium nanodomains, low-mobility buffer, Ca-sensor activation, traveling waves, imaging

## Abstract

A concept of Ca^2+^ nanodomains established in the cytoplasm after opening single-calcium channels helps mechanistically understand the physiological mechanisms of Ca^2+^ signaling. It predicts standing gradients of cytoplasmic free Ca^2+^ around single channels in the plasma membrane. The fate of bound Ca^2+^ attracted much less attention. This study aimed to examine the profiles of Ca^2+^ bound to low-mobility buffers such as bulky Ca^2+^-binding proteins. The solution of non-linear PDEs for an immobile buffer predicts fast decay of free [Ca^2+^] from the channel lumen and the traveling wave for bound Ca^2+^. For low-mobility buffers like calmodulin, the calculated profiles of free and bound Ca^2+^ are similar. Theoretical predictions are tested by imaging 1D profiles of Ca^2+^ bound to low-mobility fluo-4-dextran. The traveling waves of bound Ca^2+^ are observed that develop during the opening of single channels. The findings tempt to propose that Ca^2+^ signaling may not be solely related by the absolute free [Ca^2+^] at the sensor location, which is extremely localized, but determined by the time when a wave of bound Ca^2+^ reaches a threshold needed for sensor activation.

## 1 Introduction

Many fundamental physiological Ca^2+^-dependent processes are initiated by local [Ca^2+^] increases generated in the vicinity of the Ca^2+^ entry into the cell and dubbed as Ca^2+^ nanodomains (Eisner et al., 2023; Augustine et al., 2003; Eggermann et al., 2011). The processes attracted much attention from researchers, but their theoretical description is yet far from complete. The first important result was obtained by Neher (1986), who considered steady-state Ca^2+^ distributions in excess buffer approximation, EBA.

The EBA formalism has been developed to explain the different effects of the Ca^2+^ buffers BAPTA and EGTA in modulating the activity of nearby Ca^2+^-dependent K^+^-channels (blocking vs non-blocking actions, respectively). For channel activation, Ca^2+^ entering into the cytoplasm must reach the neighboring K^+^ channel. The distance between Ca^2+^ and K^+^ channels then should be smaller than the width of free [Ca^2+^] transient. BAPTA and EGTA produce Ca^2+^ nanodomains with different widths because EGTA captures Ca^2+^ apparently slower than BAPTA. The on-rate constant for Ca^2+^ binding by EGTA is assumed to be 100-fold lower than that for BAPTA and corresponds to a 10-fold bigger intrinsic space constant *r*_*o*_ (Table 1). The smaller width of Ca^2+^ nanodomains in BAPTA explains why this buffer abolishes the coupling between Ca^2+^ and Ca^2+^-dependent K-channels, whereas EGTA has virtually no influence.

**TABLE 1:** Basic definitions and model parameters.

Abbreviation	Definition	Numerical value
*B*_*o*_	Total buffer concentration	0.2 mM
*c =* [Ca^2+^]*/B*_*o*_	Normalized Ca^2+^ concentration	0.1–10
*f =* [*F*]*/B*_*o*_	Normalized free buffer concentration	0–1
*b =* [*B*]*/B*_*o*_	Normalized Ca^2+^-bound buffer concentration	0–1
*D*_*Ca*_	Ca^2+^ diffusion coefficient	200 μm^2^/s
*d = D*_*b*_ */D*_*Ca*_	Relative buffer diffusion coefficient	0–0.03
*k*_*on*_	On-rate binding constant	10^8 ^M^-1^s^-1^
*k*_*off*_	Off-rate constant	10^2^ s^-1^
*K*_*d*_	Ca^2+^ buffer dissociation constant	10^−6 ^M
*γ = k*_*off*_/*k*_*on*_ *B*_*o*_	Normalized dissociation constant	0.002
*i*	Single-channel current	0.1–1 pA
*A*	Normalized Ca^2+^ concentration at the channel lumen	0.1–1
*τ = 1/k*_*on*_ *B*_*o*_	Intrinsic time constant	50 μs
*r*_*o*_ *= √D*_*Ca*_ */B*_*o*_ *k*_*on*_	Intrinsic space constant	0.1 μm
*τ*_*D*_ = *x* ^*2*^ */2D*_*Ca*_	Diffusional time constant	2.5 ms

The seemingly slow calcium binding by EGTA has been previously explained (Mironova and Mironov, 2008; Mironov, 2019). Briefly, by dissolution, EGTA forms five species at different proportions in the absence of Ca^2+^. A doubly protonated EGTA (H_2_EGTA^2-^) is dominant at physiological pH. It cannot bind Ca^2+^ efficiently because *K*_*d*_ *=* 4 M (Smith et al., 1984) indicates extremely low affinity to Ca^2+^. Another form, HEGTA^3-^, has *K*_*d*_ *=* 5 µM and thus has high affinity, but in 10 mM EGTA at normal pH, [HEGTA^3-^] = 0.1 mM, i.e., 100 times smaller than nominal EGTA. A calcium ion is captured by the first buffer molecule it meets in the cytoplasm, with the on-rate close to the diffusion limit, *k*_*on*_ ≈ 10^8^ M^-1^s^-1^. Because the rate of Ca^2+^ binding is *k*_*on*_ [Buffer], an apparent 100-fold difference in *k*_*on*_ values for BAPTA and EGTA simply reflects the ratio [BAPTA]/[HEGTA^3-^] = 100. EGTA has the important property of slowly accommodating Ca^2+^ after exchanging it for protons. The equilibrium between different EGTA forms is established slowly. This explains why EGTA minimally disturbs fast local calcium transients but, at the same time, is effective in preventing calcium overload, e.g., in whole-cell recordings. On a longer time scale, EGTA virtually eliminates deleterious long-lasting increases in cytoplasmic calcium. The same considerations may be applied to another “slow” Ca^2+^ buffer, parvalbumin, which also has to free the binding sites from Mg before capturing Ca^2+^.

Ca^2+^ unbinding from the buffer is neglected in EBA because the reverse reaction is very slow. Therefore, Ca^2+^ nanodomains established during channel opening quickly disappear after channel closure. The measurements of local Ca^2+^ increases generated by single Ca^2+^ channels in the oocytes, dubbed as sparklets (Demuro and Parker, 2006), underline this notion. Despite the undisputed importance in presenting Ca^2+^ profiles around single channels, the EBA model is oversimplified.

Another limiting concept in describing Ca^2+^ behavior in the cytoplasm is rapid buffer approximation (RBA). It presumes fast equilibration between Ca^2+^ and the buffer (Neher and Augustine, 1992; Wagner and Keizer, 1994; Pape et al., 1995; Naraghi and Neher, 1997; Smith et al., 2001; Mironova and Mironov, 2008; Mironov, 2019). The corresponding equations have been known for decades (Crank, 1979) and have been successfully applied to describing global Ca^2+^ transients in bulk cytoplasm. The basic parameters in RBA are the cytoplasmic Ca^2+^-buffering capacity and apparent Ca^2+^ diffusion coefficient. The former depends on the free [Ca^2+^] level, buffer concentration, and affinity to Ca, and the latter also includes the buffer diffusion coefficient (Neher and Augustine, 1992; Wagner and Keizer, 1994; Pape et al., 1995; Naraghi and Neher, 1997; Smith et al., 2001). The current status of RBA and EBA, with numerous examples of applications in physiology, was recently surveyed in a comprehensive review (Eisner et al., 2023).

In EBA, the diffusion of the buffer is completely neglected (the steady-state Ca^2+^ gradients are dependent only on *D*_*Ca*_ through *r*_*o*_). In RBA, the buffer diffusion coefficient *D*_*b*_ is treated implicitly and included in the apparent Ca^2+^ diffusion coefficient. It is still imperative to better understand the role of buffer motility because the imaging of Ca^2+^ with common organic indicator dyes may not correctly deliver actual changes in cytoplasmic [Ca^2+^] needed to understand fundamental physiological events. Ca^2+^ signaling is mostly examined using synthetic buffers and indicators (EGTA, BAPTA, fluo-X, *etc.*). They all have diffusion coefficients compatible with that of Ca^2+^, justifying the application of EBA. In physiology, putative Ca^2+^-binding proteins have order of magnitude smaller diffusion coefficients (Sanabria et al., 2008; Schwaller, 2010; Matthews and Dietrich, 2015). In order to reproduce the *in vivo* situations, it is more consistent to employ genetically encoded sensors that are also bulky proteins (Mironov et al., 2009; Lin and Schnitzer, 2016). The changes in their fluorescence can be expected to depict physiologically relevant cytoplasmic Ca^2+^ signals better. However, care should be taken in choosing a probe with proper fast reaction time as the changes in fluorescence may reflect conformational kinetics to achieve the fluorescent state of the protein and may not reflect the “true” kinetics of local Ca^2+^ increase (Lock et al., 2015).

The actual free Ca^2+^ levels around a single channel are of extreme importance but not yet directly measured. The problem is that when free [Ca^2+^] is smaller than the concentration of cytoplasmic buffers and/or their affinity, the assumptions of both EBA and RBA are correct. However, the theoretical estimates (Mironov, 1990; Mironov, 2019) and experiments (Tay et al., 2012; Tadross et al., 2013) indicate too high calcium levels at the channel lumen. The local [Ca^2+^] may be around 1 mM, which obviously exceeds the buffer concentration (0.2 mM is a typical value). The considerations are substantiated by a current look at synaptic transmission. This process is triggered by Ca^2+^ binding to the synaptotagmin sensor (Syt). The Ca^2+^–Syt dissociation constant is in the range of *K*_*Syt*_ = 40–100 μM, as compiled from different studies (Bornschein and Schmidt, 2019). Half-activation of the sensor occurs at [Ca^2+^] = *K*_*Syt*_, and 90% activation is required to make synaptic transmission reliable. This is achieved at 10 *K*_*Syt*_, from which one can deduce that the local [Ca^2+^] at the secretion site should be in the range of 0.4–1 mM. This is within the aforementioned theoretical and experimental estimates.

All these considerations prompt examination of the time and concentration dependence of Ca^2+^ nanodomains in the vicinity of single channels, with a closer look at the effects of buffer diffusion. In the following section, I focus on slow diffusion of buffers, keeping in mind that putative Ca^2+^-binding proteins (and Ca^2+^-sensors) diffuse much slower than Ca^2+^. The analysis starts from the time-dependent RD problem for the immobile buffer, which has an analytical solution. It predicts that Ca^2+^ gradients build up relatively fast around a single channel, and the profile of the Ca^2+^-bound buffer corresponds to the traveling wave. Within a few milliseconds, such waves can propagate over 1 μm, a typical dimension for central synapses. The case is then extended to introduce the slow motility of the buffer (*D*_*b*_ *<< D*_*Ca*_) to reproduce the situation *in vivo*. For low-mobility buffers, the patterns of bound Ca^2+^ are found to be similar to those of immobile buffers. To test theoretical predictions, the 1D-distributions of Ca^2+^ in thin pipettes were examined. Ca^2+^ influx was induced by a single α-synuclein channel in inside-out patches from hippocampal neurons. Incoming Ca^2+^ was captured by a low-mobility buffer (fluo-4-dextran). 1D distributions of bound Ca^2+^ agreed well with theoretical predictions. The main conclusion of the study is that although free Ca^2+^ profiles are well-localized to the lumen of single Ca^2+^ channels, the bound species may spread out, delivering message(s) to activate distant targets.

## 2 Methods

### 2.1 Ethical approval

All mouse experiments were approved and performed in accordance with the guidelines and regulations by the local authority, the Lower Saxony State Office for Consumer Protection and Food Safety (Niedersächsisches Landesamt für Verbraucherschutz und Lebensmittelsicherheit), and executed at the Georg August University, Göttingen. Briefly, all animals had free access to the shelter and to water and food, and every effort was made to minimize animal suffering and the number of animals used. For removal of tissues, the animals were deeply anesthetized by CO_2_ inhalation at a fixed concentration and rapidly killed by cervical dislocation.

### 2.2 Cell preparations and solutions

Cultured hippocampal neurons were obtained from neonatal mice (NMRI, P3–P6), as described previously (Mironov, 2015). During the experiments, the coverslips with cells were mounted in a recording chamber that was continuously superfused at 34°C.

### 2.3 Patch-clamp and imaging

The electrophysiology and imaging in cultured hippocampal neurons are performed as described previously (Mironov, 2015; Mironov, 2019). Bath and pipette solutions contain 30 mM Tris buffer (pH 7.4) and 154 mM NaCl or 88 mM CaCl_2_. The solutions have an osmolality of 305–315 mosmol/l. Fluo-4 and Fluo-4 dextran (M.W. 30 kDa) were obtained from Thermo Fisher (Osterode, Germany). Both dyes were used at a concentration of 0.3 mM. Fluo-4 dextran was dissolved at 1% concentration. Dextran (10 kDa) has a diffusion coefficient of *D* = 15 μm^2^/s (Shuai and Parker, 2005), which is close to the measured value for calmodulin, *D* = 10 μm^2^/s (Sanabria et al., 2008). Given *D*_*Ca*_ = 200 μm^2^/s, both values are close to the relative diffusion coefficients *d* = 0.03 (Mironova and Mironov, 2008) used in this study (Table 1).

Patch electrodes were pulled from borosilicate glass (1.4 mm o. d., WPI, Berlin, Germany). The pipettes have a small bore (<0.1 µm) and a long shank (≈5 mm), with a resistance of ≈40 MOhm. Coverslips with the hippocampal neurons are placed on the microscope stage. The cells are patched, inside-out patches are excised, and synuclein (α-Syn) channels are forced to incorporate into the membrane (Mironov, 2015). The horizontally oriented pipette is then lowered to the bottom to enter the TIRF illumination layer (≈200 nm above the coverslip).

For imaging, an upright microscope (Axioscope 2, Zeiss) is used with a ×63 objective lens (N. A. 1.4). The fluorescence is excited by 488 nm light from an SLM Diodenlaser (Soliton, Gilching, Germany). The laser beam is delivered from below through a prism (TIRF Labs, Inc. Cary NC) at an angle appropriate to evoke TIRF. The emission (525 nm) passes through a dichroic mirror filter at 535 nm. The images are captured by using a cooled CCD camera, iXON Ultra 888 EMCCD (ANDOR, Oxford Instruments), operated under ANDOR software. The line-scan mode (512 × 2 pixels at 12-bit resolution) has an acquisition time of 0.1 ms. Because the fluorescence of fluo-4 increases >10-fold after Ca^2+^ binding, this minimizes out-of-focus effects that appear in imaging of living cells. The cell-free experimental configuration also provides good control of the solutions bathing the patch.

Single-channel events were recorded in 12 patches obtained from different neurons with the pipettes filled with Fluo-4-dextran solution. Eight patches with synuclein channels were recorded with Fluo-4 in the pipette. The episodes of channel opening were collected at different holding potentials. They were analyzed offline to examine the dependence of bound Ca^2+^ distributions upon single-channel Ca^2+^ flux and its duration.

### 2.4 Basics of the reaction–diffusion equation

To start with, consider Ca^2+^ diffusion from a single channel in the presence of a single buffer as a system of two partial differential equations (PDE)

$$\begin{array}{c} \partial \left[{\text{Ca}}^{2+}\right]/\partial t\;=D_{Ca}\Delta \left[{\text{Ca}}^{2+}\right]–\;k_{on}\;\left[{\text{Ca}}^{2+}\right]\left[F\right]+k_{off}\left[B\right],\\ \;\partial \left[F\right]/\partial t=D_{b}\Delta \left[F\right]\;–\;kon\;\left[{\text{Ca}}^{2+}\right]\left[F\right]+k_{off}\left[B\right]. \end{array}$$ (1)

Here, *Δ* stands for the Laplacian; [Ca^2+^], [*F*], and [*B*] are the concentrations of calcium and buffer in free and Ca^2+^-bound forms, respectively; *k*_*on*_ and *k*_*off*_ are the rate constants of calcium-binding to and dissociation from the buffer. The ratio *k*_*off/*_ *k*_*on*_ *= K*_*d*_ is the dissociation constant of the buffer and measures its affinity to calcium. *D*_*Ca*_ and *D*_*b*_ are the diffusion coefficients for calcium and buffer, respectively. For simplicity, the equations are set for a single buffer, but generalization for multiple buffers is straightforward, as discussed below. The definitions and typical parameters are summarized in Table 1.

In the following derivations, all concentrations are normalized by dividing by the *total* buffer concentration *B*_*o*_. Equation 1 is then written as

$$c_{t}=\Delta c\;–\;cf+\gamma \left(1\;-\;f\right),$$ (2a)

$$f_{t}=d\Delta f\;-\;cf+\gamma \left(1\;–\;f\right),$$ (2b)

where the subscript denotes the time derivative, *c =* [Ca^2+^]*/B*_*o*_ and *f =* [*F*]*/B*_*o*_ are the normalized concentrations of free calcium and free buffer, respectively; *d = D*_*b*_ */D*_*Ca*_ is the relative diffusion coefficient, and *γ = k*_*off*_/*k*_*on*_ *B*_*o*_ *= K*_*d*_ */B*_*o*_ = 0.002 is the normalized dissociation constant. The last term on the right-hand side in Eq. 2a describes Ca^2+^ dissociation, and *b = 1–f* is the normalized concentration of the Ca^2+^-bound buffer. The dimensionless times and distances are defined as *τ = 1/k*_*on*_ *B*_*o*_ = 50 μs and *r*_*o*_ *= √D*_*Ca*_ */B*_*o*_ *k*_*on*_ = 0.1 μm, respectively. These intrinsic constants of Ca^2+^-buffering (Table 1) are calculated for diffusion-limited *k*_*on*_ = 10^8^ M^-1^s^-1^ and *B*_*o*_ = 0.2 mM, typical values for cytoplasmic calcium-binding proteins, and *D*_*Ca*_ = 200 μm^2^/s.

The boundary condition is specified by assuming the constant flux through the open channel

$$c_{r}=-i/2\pi zR^{2}D_{Ca}F,$$ (3)

where *i* is a single-channel current, *F* is the Faraday constant, and *R* is the exit radius (*R =* 0.5 nm, (Malasics et al., 2009)). For radial diffusion, it is useful to transform *c = u/r*, which replaces the Laplacian *Δ* = *1/r* ^*2*^ *d* (*r* ^*2*^ *dc/dr*) with the second derivative *Δ* = *d* ^*2*^ *u/dr* ^*2*^. The boundary condition transforms into

$$Ru_{r}\;–\;u\;\approx \;-u=-AR,$$ (4)

where *A* is the Ca^2+^ concentration at the channel lumen. Because *R* is very small, the first term can be neglected. For *i =* 0.1 pA, the constant is *A =* 0.7 mM.

The linearized version (EBA) assumes a fast irreversible binding of Ca^2+^ to buffer. This sets *f* = 1 and *γ* = 0 in Eq. 2a and leads to a simple ordinary differential equation (ODE) of the second order

Δc=c, (5)

which has an exponential–hyperbolic solution

$$c=\left(A/r\right)\exp\left(-r/r_{o}\right),$$ (6)

The solution of the transcendental Equation 6 is given by the Lambert function defined as *W = exp* (*-W*) (Olver et al., 2011). The reference point may be *r* = 0.57 *r*_*o*_ for *c = A.* In the 1D case, the *1/r* factor is absent, and *r* = 0.37*r*_*o*_.

### 2.5 Numerical solution of reaction–diffusion equations

The PDEs derived in the text below are solved with Du Fort–Frankel numerical integration (Du Fort and Frankel, 1953). Its performance is better than that of the Crank–Nicolson algorithm and unconditionally stable. For discretization of diffusion equation *c*_*t*_ *= Dc*_*xx*_, the concentrations at times *t*_*n*_ *= nΔt* and space points *x*_*m*_ *= mΔx* are needed. The following parameters are calculated

$$d_{1}=\left(1\;-\;\alpha \right)/\left(1+\alpha \right),d_{2}=\alpha /\left(1+\alpha \right),\text{with }\alpha =2D\Delta t/\Delta x^{2},$$ (7)

and the reaction–diffusion (RD) equation is then solved explicitly at each mesh point as

$$c_{m}^{n+1}=d_{1}\;c_{m}^{n-1}+d_{2}\;\left(c_{m+1}^{n}+c_{m-1}^{n}\right)-\;\Delta t\;c_{m}^{n}\;f_{m}^{n}.$$ (8)

The last term is the reaction term (-*cf*) specified by respective terms in the PDEs derived below.

## 3 Results

### 3.1 Immobile buffer

Ca^2+^ diffusion in the presence of a single immobile buffer is described by the PDE system as

$$c_{t}=\Delta c\;–\;cf,$$ (9a)

$$f_{t}=-cf\;.$$ (9b)

For the initial concentration of free buffer *f*_*o*_ *= 1*, integration of the second equation gives

$$f=f_{o}e^{-y},$$ (10)

where a new variable,

y=∫cdt, (11)

the integral of Ca^2+^ concentration, is introduced. Integration of Eq. 9a then gives a non-linear PDE

$$y_{t}=\Delta y\;-\;1+e^{-y},$$ (12)

with the boundary condition *y*_*R*_ *= tc*_*R*_. Equation 12 has no general solution even in the 1D case except a self-similar particular solution in moving coordinates *z = ax–bt* (Polyanin and Zaitsev, 2004).

**FIGURE 1:**
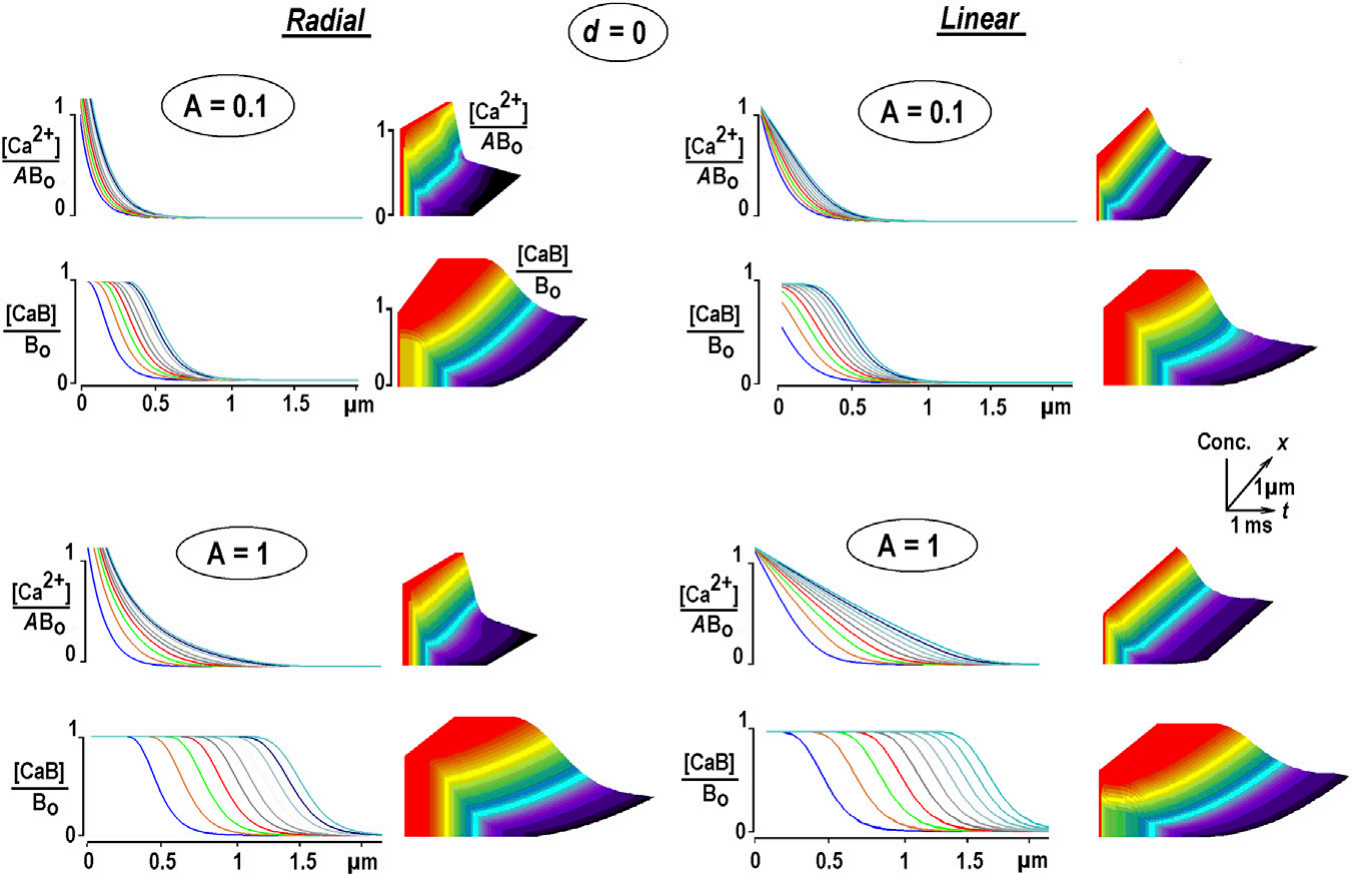
Changes in free and bound Ca^2+^ in the presence of an immobile buffer. The solutions are obtained from Eq. 12. Differently colored curves are 0.25 ms apart. Time and space scales are the same in all panels. The concentrations of free and bound Ca^2+^ [CaB] are normalized to the total buffer concentration, *B*_*o*_. To compare the waveforms of transients of free [Ca^2+^], it is additionally divided by the concentration of Ca^2+^ at the channel lumen (*A*). Radial Ca^2+^ increases near the channel are cut from above because they are too big due to the hyperbolical factor *1/r* that appears in radial diffusion, Eq. 6. At constant Ca^2+^ influx, the Ca-bound buffer spreads out, which mirrors a depletion of the free buffer. The waves have a *tahn*-like form, a hallmark of many RD systems (Polyanin and Zaitsev, 2004). The increment between the curves decreases with time, indicating a decrease in instantaneous velocity. Each panel is complemented with 3D plots on the right, with the axes defining time, space, and relative concentrations, respectively (the scaling is indicated in the inset in the middle). The concentrations in the panels are plotted on the *z*-axis, and increases are represented by warmer colors, with time running from left to right along the *x*-axis.

Eq. 12 (Smith et al., 2001) was solved numerically using the Du Fort–Frankel algorithm described in above. Figure 1 shows the calculated radial (3D) and 1D profiles for free Ca^2+^ and Ca^2+^-bound buffers for different Ca^2+^ fluxes. They are coded as relative Ca^2+^ levels at channel lumen *A* = [Ca^2+^]/*B*_*o*_. It is seen that [Ca^2+^] decays rapidly from the entry site. In the 3D case, [Ca^2+^] is very big due to the hyperbolic factor *1/r*, intrinsic in radial symmetry, Eq. 6 (Mironov, 2019). Large free Ca^2+^ values strongly violate the EBA assumption that [Ca^2+^]<<*B*_*o*_. The spatial profiles of the Ca^2+^-bound buffer expand with time as traveling waves. They appear already for a very small relative Ca^2+^ flux *A* = 0.01 and widen with increasing *A* (Figure 1). The increment between subsequent curves decreases with time, indicating a decrease in wave speed.

A simple analytical argument helps understand the appearance of traveling waves. Free Ca^2+^ approach fast exponential–hyperbolic steady-state solution given by Eq. 4 in EBA. Under such conditions, the bound Ca^2+^ is obtained from Eq. 9b as

$$\left[{\text{Ca}}^{2+}-bound\right]=b=1-\;f=1\;-\exp\left[-At/r_{o}^{.}\exp\left(-r/r_{o}\right)\right].$$ (13)

The expression describes the traveling wave spreading out according to the time constant proportional to the Ca^2+^ level at channel lumen, *A*.

Another interesting feature emerges when we identify, for a moment, the bound buffer with an immobile Ca^2+^-sensor (McMahon and Jackson, 2018). The temporal and spatial patterns of sensor activation should then be given by the profiles for the Ca^2+^-bound buffer, as shown in Figure 1. From this, one may deduce that a crucial factor in the Ca^2+^-mediated functional response may be the time when a sensor at a given location is saturated with Ca^2+^. A suggestion with such a role of bound Ca^2+^ underlines a statement that “messages diffuse faster than messengers” (Pando et al., 2006) coined by simulating the diffusion of particles in the presence of traps as applied for GFP-tagged glucocorticoid receptors in the nuclei of mouse adenocarcinoma cells. The mathematical formulation of the problem resembles that considered in this study.

Both the 3D and 1D cases of Ca^2+^ diffusion are physiologically relevant because they present Ca^2+^ diffusion defined by specific geometric constraints. For radial diffusion, Ca^2+^ spreads out in a semi-infinite cytoplasm free from intracellular organelles of macromolecular aggregates that may hinder diffusion. 1D diffusion may imitate Ca^2+^ diffusion through a tortuous pathway that extends from the point of Ca^2+^ entry to the sensor. A well-known 1D example is the Ca^2+^ waves in neuronal dendrites (Augustine et al., 2003; Mironov, 2008). Of note, in linear models, the 3D and 1D solutions are related: the former is obtained from the latter by simple division of the solution by the distance from the channel lumen. The application of such transformation is shown in Supplementary Figure SB.

A general conclusion from this section is that bound Ca^2+^ distribution in the presence of an immobile buffer is dynamic rather than static. A simple time-dependent model shows inherent contradictions with the linear EBA model: (i) free buffer is not in excess even for small Ca^2+^ fluxes (*i* < 0.1 pA), (ii) not constant, and (iii) shows the time- and space-dependent changes in bound Ca^2+^ that mirror buffer depletion. The conclusions are also valid for low-mobility buffers, as examined in the following section.

### 3.2 Slowly moving buffer

The results in the previous section are extended below to the case of a slowly moving buffer. Eq. (2) is now given as

$$\begin{array}{l} c_{t}=\Delta c\;–\;cf,\\ f_{t}=d^{.}\;\Delta f\;-\;cf. \end{array}$$ (14)

**FIGURE 2:**
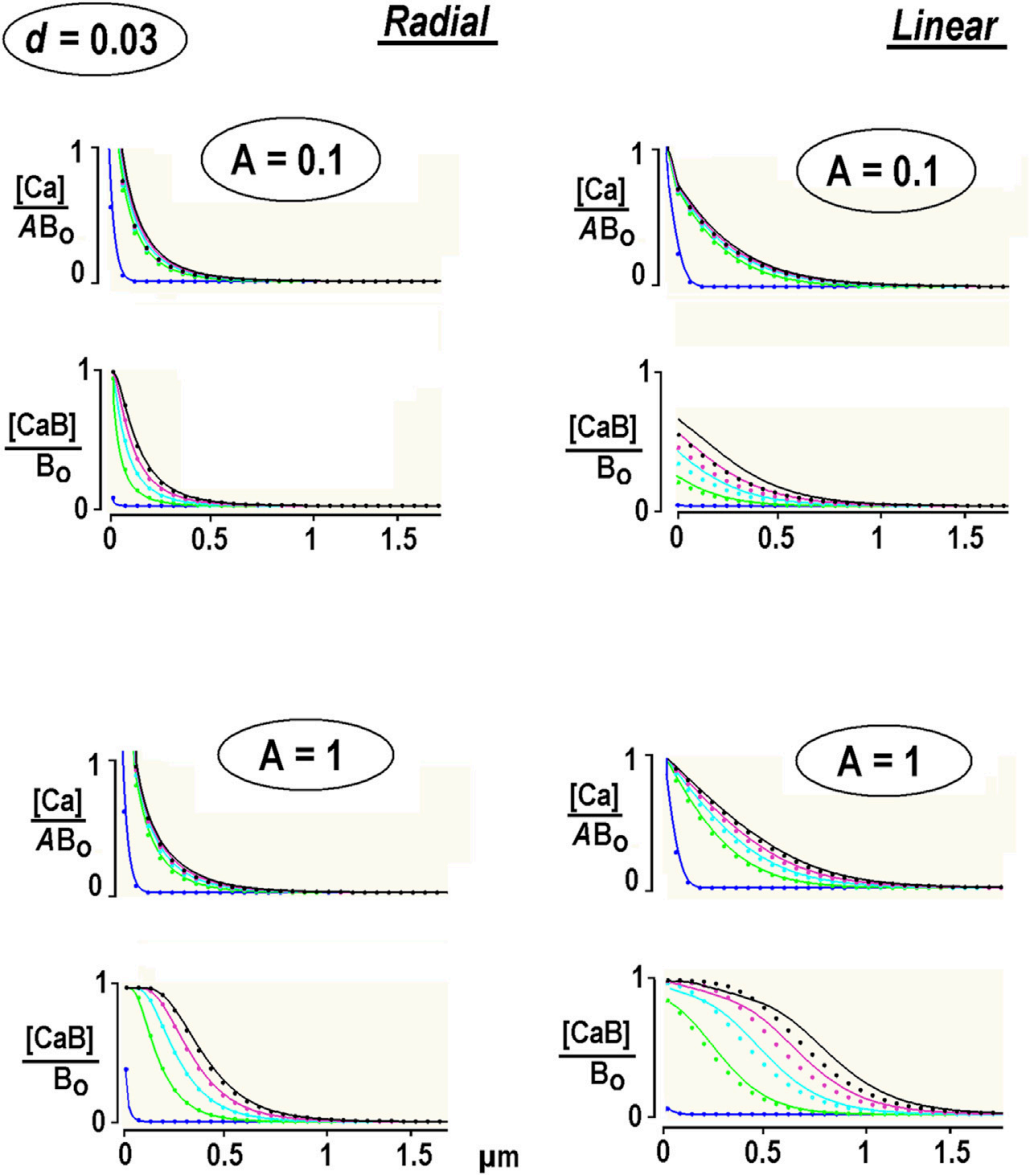
Diffusion of free and bound Ca^2+^ in the presence of a low-mobility buffer. The concentration profiles are obtained from the non-linear PDEs for low-mobility buffer (smooth curves, Supplementary Appendix SA1) and immobile buffer [dots, Eq. 12]. The first curve is taken at 0.1 ms, and the interval between the following curves is 0.25 ms. Each panel presents the results for different relative Ca^2+^ concentrations at the channel lumen, *A* = [Ca^2+^]/*B*_*o*_. Free [Ca^2+^] is also divided by *A* to compare the waveforms.

The system was solved in Supplementary Appendix SA1. The calculations presented in Figure 2 were made for calmodulin, the relative diffusion coefficient of which is set to *d* = 0.03. This is compatible with a relative diffusion coefficient *d* = 0.07 for an endogenous “immobile” buffer in chromaffin cells (Neher and Augustine, 1992). The radial and linear Ca^2+^ profiles resemble the data obtained for the immobile buffer (the dots in Figure 2). A close relationship validates expansion on a small parameter *d* applied in deriving the solution of Eq. B1 in Supplementary Appendix SA1. It also indicates that immobile buffer approximation serves as a good approximation for slowly moving buffer.

Free Ca^2+^ approached the steady state quickly, but the bound Ca^2+^ was never stationary. [CaB] waves became denser with time. The speed of changes in free and bound Ca^2+^ changes with time and can be characterized by the instantaneous velocity. This was estimated as the *x*-distance between the half-amplitude points divided by the time increment between the respective curves. For linear diffusion with small Ca^2+^ flux (*A* = 0.1, the right panels in Figure 2), it subsided from 56 to 3 μm/s, as estimated at 0.1 and 5 ms after the pulse. The velocity changed at the same time points from 78 to 18 μm/s for bigger Ca^2+^ fluxes (*A* = 1). In contrast, the bound Ca^2+^ “moved” much faster. For *A* = 0.1, the velocities at 0.1 and 5 ms after beginning the pulse were 84 and 46 μm/s, and for *A =* 1, they were 107 and 85 μm/s, respectively. For the radial diffusion (Figure 2, the left panels), the values are around 40% smaller. This can be explained by the role of the damping hyperbolic factor (1/*r*). The 1D values are in the range for Ca^2+^ waves measured in neuronal dendrites (Augustine et al., 2003; Mironov, 2008). The data thus demonstrate that the bound Ca^2+^ apparently “moves” much faster than free Ca^2+^ and overtakes it within a few milliseconds after switching on Ca^2+^ influx.

### 3.3 Patch-clamp and imaging

**FIGURE 3:**
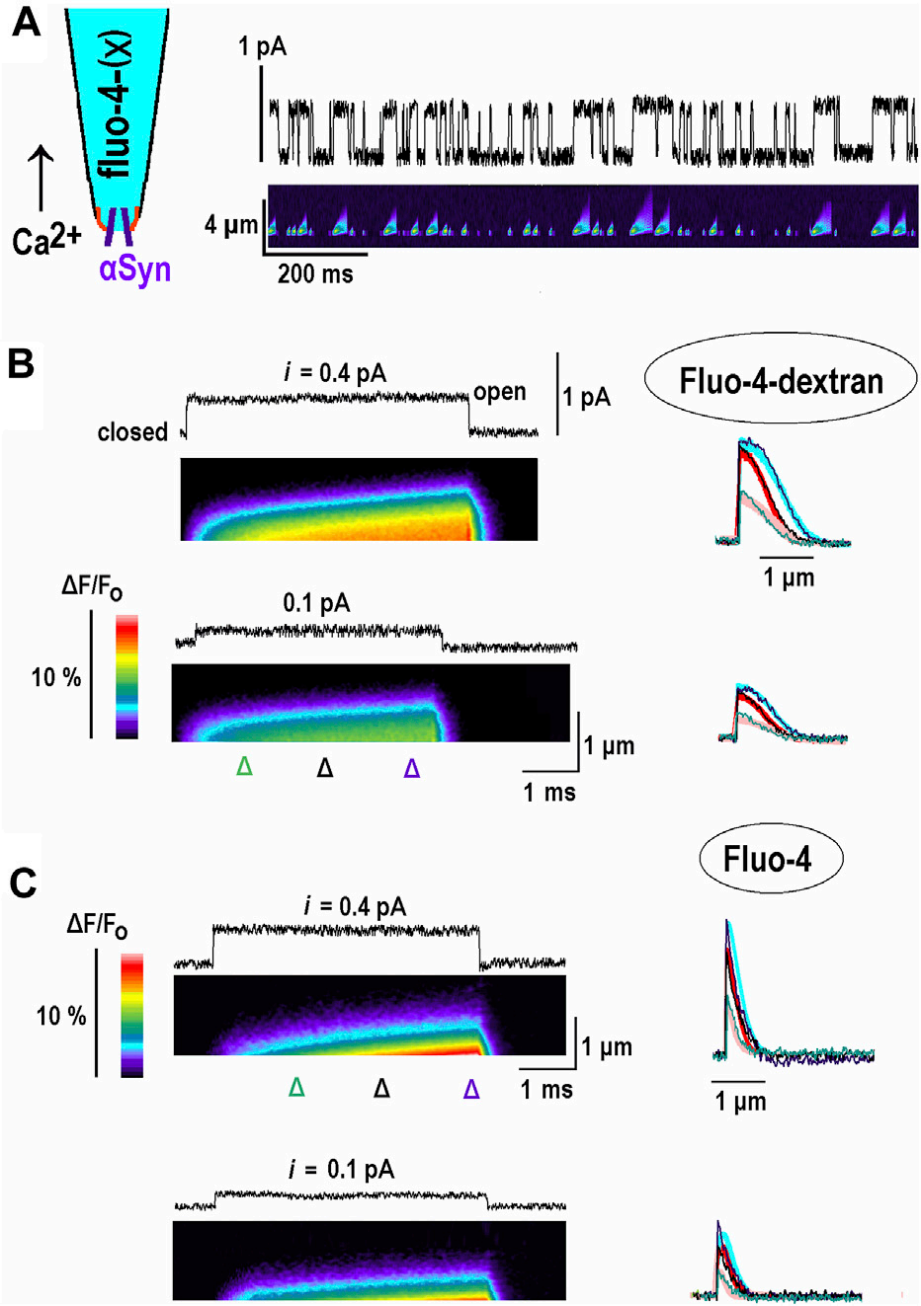
Changes in fluorescence of Ca^2+^-bound buffer due to opening of single α-synuclein channels in inside-out patches. α-Synuclein (α-Syn) channels are incorporated into inside-out patches excised from cultured hippocampal neurons (see Methods and Mironov (2015); Mironov (2019)). The pipette solution contained fluo-4-dextran **(B)** or fluo-4 **(C)** in 154 mM NaCl, and the bathing solution contained 88 mM CaCl_2_. Opening of channels in this configuration (the inset in the left upper corner in **(A)** induced “outward” Ca^2+^ current and local increases of Ca^2+^ bound to indicator dyes. During 1-s long pulses, at different potentials from 10 to 20 openings were observed. The patterns of CaB fluorescence were stereotypic and scaled by duration of opening, which ranges from 3 to 20 ms, and the single channel current. Following the return to the closed state, the fluorescence decreased fast due to Ca^2+^ diffusion and reaction with remaining free buffer molecules. **(A)**. Experimental configuration and simultaneous recording of channel activity at +80 mV (upper trace) and fluorescence of Ca^2+^ bound to fluo-4-dextran (lower kymograph). The record is a sample episode of 1-s-long excursions from 0 mV. **(B)**. Each panel shows a single-channel current and the fluorescence of Ca^2+^ bound to Fluo-4 dextran (kymographs). They present relative changes in fluorescence (background subtracted, calibration bar on the left). The panels depict fluorescence measured along a line on the line-scan of the *y*-axis (distance from the tip), with time running from left to right along the *x*-axis. Increased normalized fluorescence (*F/F*_*o*_) corresponded to an increase in bound Ca^2+^ (warmer colors). The line scans made at time points are indicated by triangles below the kymograph. They are presented on the right and indicate a spread out of bound Ca^2+^. Experimental traces are overlaid upon the thick theoretical curves, which were calculated as described in the Results section and show good agreement. **(C)**. The same protocols were repeated with Fluo-4 in the pipette. Note that in the case of the high-mobility buffer both free and bound [Ca^2+^] built up the stationary gradients fast.

In order to test theoretical predictions, I sought for experimental proof-of-principle and aimed to obtain the 1D profiles of the Ca^2+^-bound buffer. They were imaged after opening of single channels, and bound Ca^2+^ was monitored within a thin pipette filled with fluo-4(X) indicator dyes (inset in Figure 3A). α-Synuclein forms in the plasma membrane of hippocampal neurons’ Ca^2+^-permeable channels (Mironov, 2015). A single channel in the inside-out patch influx was used to generate controllable “outward” Ca^2+^ influx into the patch pipette. The data shown are representative of recordings made in 12 patches for fluo-4-dextran and in eight patches for fluo-4.

This 1D experimental system for examination of CaB waves has several advantages: (i) the number of pixels to collect is equal to *N*, not to *N* ^*3*^ as in the 3D case, which allows faster measurements; (ii) 1D profiles are more extended because they do not include a “hyperbolic factor”, 1/*r*, a distance from the source (Figures 1, 2); (iii) the openings are longer than generated by voltage-dependent Ca^2+^ channels, which open normally less than for a couple of milliseconds, which allows more room for the waves to develop.

The channels in the inside-out patch show stable activity for about 20 min. The patch was held at 0 mV, where the current was 0, and the potential was periodically shifted for 1 s. Between 10 and 20 openings are observed during the pulse (Figure 3A). Changes in fluorescence in response to channel opening were stereotypic and depended only on opening duration and single-channel current. The distribution of bound Ca^2+^ expanded with time and formed a traveling wave (Figure 3B). After returning to the closed state, the fluorescence decreased fast due to Ca^2+^ diffusion and reaction with free buffer molecules. In the 1D case, the diffusional time constant can be estimated as *x* ^2^/2*D*_Ca_ = 1 μm^2^/(2.200 μm^2^/s) = 2.5 ms. When only 10% free buffer is available, the reaction time constant is small, 1/*k*_*on*_ *B*_*o*_ = 1/(2 × 10^−5 M^) (10^8^ M^-1^s^-1^) = 0.5 ms, which promotes fast decay of bound [Ca^2+^]. The vertical cross-sections of the kymograph in the rightmost panel in Figure 3B show the relative fluorescence in time and space and indicate a spread out of bound Ca^2+^. Experimental traces are overlaid upon thick theoretical curves calculated using the model described above and plotted in Figure 2. Both sets of data show a good agreement.

The experiments were also carried out with Fluo-4-filled pipettes. The distribution of bound Ca^2+^ was narrower and quickly attained the steady state. This is expected as Fluo-4 has a diffusion coefficient of approximately 100 μm^2^/s (Mironov, 2019), which is close to that of Ca^2+^. These experiments may be treated within the EBA framework by setting the relative diffusion coefficient in Eq. (2) to *d* = 1. The solution is obtained in Supplementary Appendix SB1 and used to plot the thick traces in Figure 3C. The theoretical predictions show good correspondence with experimental traces.

## 4 Discussion

Ca^2+^ nanodomains established in the cytoplasm in the vicinity of a single calcium channel represent an accepted paradigm to explain secretion during synaptic transmission (Eisner et al., 2023; Augustine et al., 2003; Eggermann et al., 2011; Südhof, 2017). As discussed in the Introduction, the existence of local Ca^2+^ gradients is based upon the simplest (and widely used) linearized model, which uses the excessive buffer approximation, EBA (Neher, 1986). It assumes irreversible Ca^2+^ binding and a constant buffer concentration and does not consider buffer diffusion (or implicitly assumes that the buffer mobility is similar to that of Ca^2+^). The neglect of possible saturation, and thus depletion of free buffer, may yet invalidate a simple linearized model.

The aim of the study is to examine the time-dependent Ca^2+^ changes around an open Ca^2+^ channel. The non-linear RD problem of Ca^2+^ diffusion in the presence of a low-mobility buffer has the rationale that putative Ca^2+^-binding species are bulky proteins whose diffusion coefficients are smaller than *D*_*Ca*_ by an order of magnitude. The analysis starts with the case of an immobile buffer. The solution of the non-linear Eq. (9) shows that [Ca^2+^] transients decay and stay localized to the open channel, similar as in EBA. More intriguing are the profiles of bound Ca^2+^, which forms the traveling waves (Figure 1). This result follows directly from EBA, as given by Eq. (9). This obviously contradicts the assumption about the constant concentration of free buffer that should mirror the profile of bound Ca^2+^. The traveling waves of the Ca^2+^-bound buffer appear already for small Ca^2+^ fluxes. Their speed ranges from 20 to 120 μm/s, and they are faster for 1D diffusion. The velocity is high enough to reliably deliver Ca^2+^-mediated changes along submicron distances within a few milliseconds. The scales are compatible with the size of both post- and pre-synaptic endings and the duration of fast synaptic events.

The low-mobility buffer only slightly modifies the waveform of waves of bound Ca^2+^ (Figure 2). We may also consider a Ca^2+^-bound buffer as a Ca^2+^-sensor (McMahon and Jackson, 2018). Indeed, if the buffer and sensor have identical binding and diffusion characteristics, their concentration changes should be identical. EBA treats the width of Ca^2+^ nanodomains as a major determinant of Ca^2+^-mediated signals. The situation is yet dynamic, not static, and the distribution of Ca^2+^-bound species is extended in time and space. This may be crucial in Ca^2+^ signaling, in which the onset of related events may be determined by the time needed for the Ca^2+^-bound sensor at a given location to reach a threshold for activation. Of course, further reaction steps may follow Ca^2+^ binding that would expand the activation zone in time and space and correspondingly delay a physiological event. The inclusion of subsequent reaction steps in sensor activation would be possible.

The main advantage of the model is the possibility to obtain the analytical solution and predict novel buffer-mediated effects. Of course, it is still oversimplified and has several limitations worth mentioning: (a) it operates on a fast time scale (<10 ms) and neglects Ca^2+^ dissociation from the buffer, (b) it skips the effects of buffer saturation, which were examined in Mironov (2019), and (c) it considers only a single Ca^2+^ buffer. A careful analysis of the effects needs further work, but it may change the quantitative, not the qualitative, outcome of the study.

For example, the cytoplasm contains multiple buffers with different properties, and the case of a single buffer is readily extended to such situations. When Ca^2+^ unbinding is neglected, the characteristic temporal and spatial scales (Table 1) can be defined through the apparent rate constant *k =* ∑*k*_*on,n*_ *B*_*o,n*_, which is a sum of all buffers present. A good guess is to assume approximately the same *k*_*on,n*_, close to the diffusion limit *k = k*_*on,dl*_. Then, the introduction of apparent *k = k*_*on,dl*_ ∑*B*_*o*_ is equivalent to the consideration of a single buffer with concentration ∑*B*_*o*_, as treated in the report. “Slow” buffers like EGTA and parvalbumin can also be included. As discussed in the Introduction, their slow Ca^2+^ binding is only apparent. This is due to the small amount of available Ca^2+^-binding sites, as the others are already occupied by protons (EGTA) and Mg^2+^ (parvalbumin). A diffusional correction (Supplementary Appendix SA1) is also linear and represented by a sum of contributions from all buffers taken with appropriate weights, i.e., the relative concentrations.

Ca^2+^-unbinding may become important for bigger *k*_*off*_ > 1,000 s^-1^ corresponding to *τ*_*off*_ < 1 ms, which becomes compatible with the channel open and closed times. The case is relevant for small organic Ca^2+^-binding species, such as citrate, lactate, and oxalate Their effects on local Ca^2+^ transients are not examined yet, but in such studies, at least two side effects should be considered. The low-affinity buffers are weak electrolytes equilibrated with neutral forms, which are membrane-permeable. Penetration into intracellular organelles such as mitochondria may influence physiological responses. Ca^2+^-binding will also displace protons from the binding sites of low-affinity buffers, resulting in fast changes in the cytoplasmic pH, which may invoke specific effects.

In summary, theoretical treatment and experimental data of Ca^2+^ diffusion in the presence of a slowly moving buffer show the spread out of bound Ca, which may play an important role in physiological events. In particular, Ca^2+^-mediated signaling may be determined by the time when a threshold level of sensor activation is reached by a wave-transported Ca^2+^-bound species.

## Data Availability

The raw data supporting the conclusions of this article will be made available by the authors, without undue reservation.
