# Supplementary material for: Bound Ca2+ moves faster and farther from single open channels than free Ca2+
Source: Front Physiol. 2023 Dec 20;14:1266120. doi: 10.3389/fphys.2023.1266120 (PMC10761531; doi:10.3389/fphys.2023.1266120)

***Appendix A.* Time-dependent solution for slow moving buffer.**

For solving the system (14) the Ca^2+^ and free buffer concentrations are expanded as *c = c + μC* and *f = f + μF,* and time-dependent buffer diffusion is considered as a perturbation. For *μ^0^* = 1 (zero order), the concentration of free buffer and Ca^2+^ are determined from Eq. (9). The first order correction (*μ^1^*) is given by the linear system

*C_t_ = ΔC – fC – cF,* (B1a)

*F_t_ = d Δf – fC – cF,* (B1b)

Using the identity in the zero order *c =* *-f_t_/f*, Eq. (9b), transferring the last term from the right-hand-side of Eq. (15b) to the left, and dividing both sides by *f,* we obtain

*F_t_/f - Ff_t_ / f^2^ =* (*F/f*)*_t_ = d Δ f / f – C _,_* (B2)

In the first order initial *Y* and *F* = 0, and the integration gives

*F = d f ∫*(*Δf/f*) *dt – fY _,_* (B3)

where *Y = ∫C dt* akin Eq. (11)*.* Eq. (15) is then

*C_t_ = ΔC – fC - cF* = *ΔC + F_t_* – *d^.^ Δf,* (B4)

Plugging into it the expression for *F_t_* derived from (16), the explicit equation for the first order correction is obtained

*C_t_ = ΔC –* (*fY*)*_t_ + c^.^f^.^Y + d*(*Δf/ f*) *^.^ f_t_ ^.^ dt,* (B5)

where identities *f_t_ =* *-cf* and *Y = ∫Cdt* for are used.

The PDE is numerically integrated using Du Fort-Frankel scheme and the results are presented in Fig. 2. The radial and linear Ca^2+^ profiles are close to the data obtained for immobile buffer (the dots in Fig. 2). A close relationship validates expansion on a small parameter *d* applied in Eq. (14) and shows that the immobile buffer approximation serves as a good approximation for slowly moving buffer.

***Appendix B.* Solution of time-dependent 'standard' excess EBA equation for equal mobilities of free Ca^2+^ and buffer.**

Eq. (2a) in the main text for free Ca^2+^ in EBA approximation reads as

*c_t_ = Δc – c*, (B1)

To solve it the Laplace transform (LT) *C = ∫e^-pt.^dt* is first performed that gives the ODE

*pC = ΔC –C*, (B2)

For *C = A* at *x = 0*, the solution is

*C =A^.^e^qx^/p,* (B3)

where *q =√*(*p + 1*)*.* LT inversion for *e^-qx^/p* is tabulated (Prudnikov et al. 1992) and reads as

*S* (*z*) *= e^-x^ erfc*(*z - √t*) + *e^x^ erfc*(*z + √t*) (B4)

where *erfc*(z) = *∫exp*(*-z^2^*)*^.^dz* is the complementary error function and z = *x/√2t* is a self-similarity variable. Using normalized definition for Ca^2+^ bound buffer *b = 1 - f*, a respective PDE is readily obtained from Eq. (2b) and reads as

*b_t_ = Δb + c*, (B5)

After LT, it becomes

*pB = ΔB – C*, (B6)

Its solution is

*B = A^.^* (*e^-qx^ - e^-Qx^*)*/p,* (B7)

where *Q = √p.* LT is readily inverted giving

*B = A^.^* (erfc(*z*) *- S*(z)), (B8)

This solution was used to generate theoretical EBA predictions shown as wide curves upon experimental traces were overlaid (in Fig. 3C).

Prudnikov, A. P., Yu. A. Brychkov, and O. I. Marichev. 1992. Integrals and Series: Inverse Laplace Transforms. Gordon & Breach Science Publishers. New York.

**Fig. B. Replotting 1D-data in radial presentation.**

Experimental data obtained in 1D-model system akin presented in Fig. 3 with Fluo-4-dextran. The images correspond to single channel currents *i* = 0.4 pA (the upper panel) and 0.1 pA (the lower panel). They are transformed into radial profiles by dividing measured relative fluorescent values by the distance from pipette tip (distance to channel). The transformation unproportionally increased fluorescence and bigger values were cut off at small distances ('white areas'). Note spreading out of bound Ca^2+^ images similar to '1D' images.


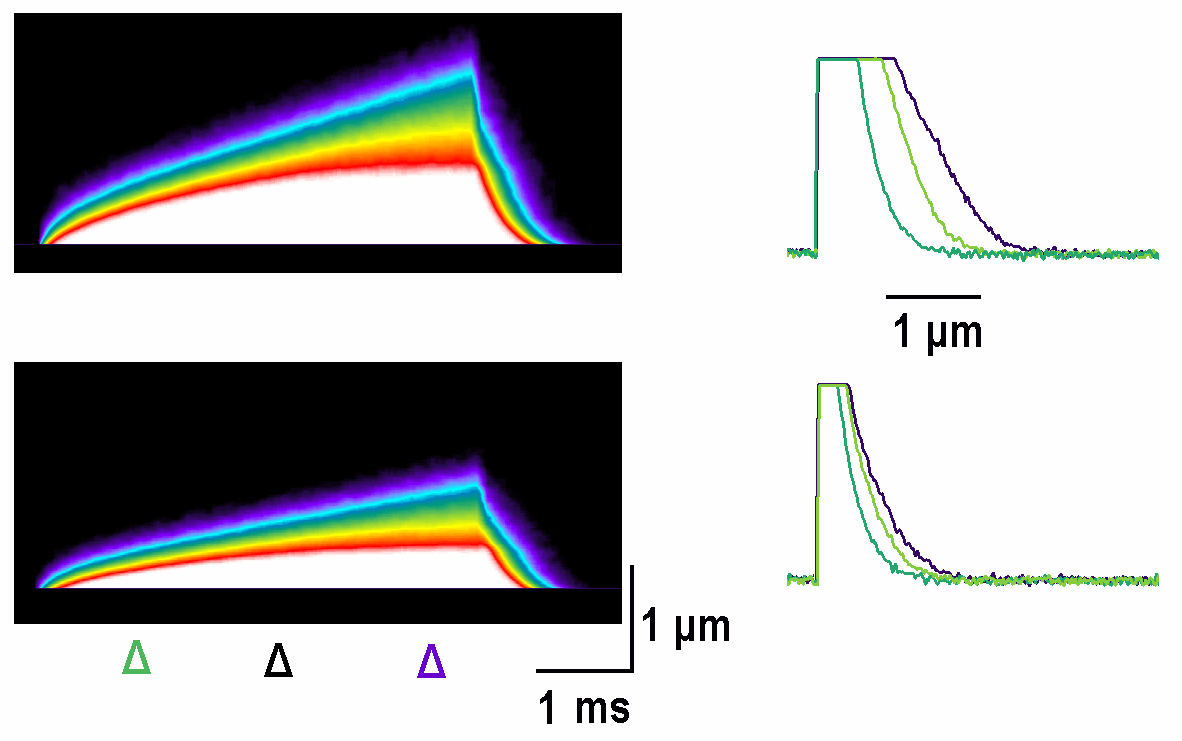

Supplement: Supplementary file 1 [file DataSheet1.docx]
